# Modeling the HIV Epidemic in Africa

**DOI:** 10.1371/journal.pmed.0020023

**Published:** 2005-01-11

The HIV epidemic is continuing to grow, year by year. According to the Joint United Nations Programme on HIV/AIDS (UNAIDS), in 2004 there were more people living with the virus than ever before, and in the same year more people than ever before died of it. So, although in the developed world HIV/AIDS is a controllable disease, one with which a treated person might expect to have a near normal lifespan, in much of the rest of the world AIDS is still a death sentence. Despite the fact that the cost of AIDS medicine has come down to around $150 per year in the developing world—a much lower cost than previously—the drugs are still unavailable to the vast majority of patients. What is more, every infected person has the chance of infecting many others. Although huge sums of money have been poured into combating HIV/AIDS—around US$4.7 billion in 2003—UNAIDS estimates this amount is less than half of what is required by 2005, and only a quarter of what will be required by 2007, to mount a comprehensive response to AIDS in low-income and middle-income countries. One of the real dilemmas, therefore, of HIV/AIDS policy is deciding whether it is better to concentrate resources on prevention of infections or on treatment of infected individuals. Each approach has ramifications for the other, as shown by the experience in some developed countries, where an increase in availability of treatment has been accompanied by an increase in risk behavior.[Fig pmed-0020023-g001]

**Figure pmed-0020023-g001:**
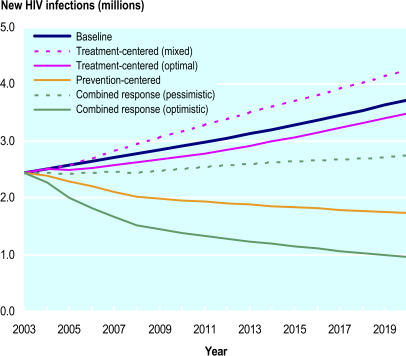
The best strategy is to combine prevention and treatment

An analysis by Joshua Salomon and colleagues in this month's *PLoS Medicine* suggests that trying to concentrate on one or the other of these alternatives is a false dichotomy, and that not integrating the two approaches could have a catastrophic effect on the global toll of HIV/AIDS by 2020. In this theoretical paper the authors analyze the epidemic in sub-Saharan Africa (where three-fourths of deaths from AIDS occur). With no change in current levels of prevention and care, it is predicted that there will be 3.7 million new HIV infections and 2.6 million adults dying of AIDS in this region each year within the next two decades. The authors predicted that concentrating on prevention alone could decrease yearly infections by half, and that concentrating on treatment could decrease yearly infections by 6%. However, combining both approaches could yield substantially greater benefits than the sum of the two alone—lowering projected new infections by 74% and projected annual mortality by half. These percentages translate into 29 million new infections and 10 million deaths averted between 2004 and 2020.

The challenge now is obviously how to put these policy suggestions into practice. The current World Health Organization treatment target of having three million people on antiretroviral therapy by the end of 2005 (the “3 by 5” objective) provides a yardstick for only one part of the equation. The authors comment that the mobilization of communities that will be needed to achieve the 3 by 5 objective should also be harnessed for prevention, and that those who teach prevention must also be allowed to get care for those infected. As the authors say, only by doing so “will we at last move from slogans to impact.”

