# Correction: Modeling the HIV Epidemic in Africa

**DOI:** 10.1371/annotation/6bfc9dda-414d-4f53-8c31-34d19bf84e4b

**Published:** 2012-03-05

**Authors:** Modeling the HIV Epidemic in Africa

An image that was published alongside another PLoS Medicine article (doi/10.1371/journal.pmed.0020024) and included in the shared PDF download here has been removed from the published PDF because of that image's copyright status. 

